# Overview and detectability of the genetic modifications in ornamental plants

**DOI:** 10.1038/s41438-019-0232-5

**Published:** 2020-02-01

**Authors:** Anne-Laure Boutigny, Nicolas Dohin, David Pornin, Mathieu Rolland

**Affiliations:** Anses, Plant Health Laboratory, Bacteriology Virology GMO Unit, 7 rue Jean Dixméras, 49044 Angers, cedex 01, France

**Keywords:** Molecular engineering in plants, Transgenic plants

## Abstract

The market of ornamental plants is extremely competitive, and for many species genetic engineering can be used to introduce original traits of high commercial interest. However, very few genetically modified (GM) ornamental varieties have reached the market so far. Indeed, the authorization process required for such plants has a strong impact on the profitability of the development of such products. Considering the numerous scientific studies using genetic modification on ornamental species of interest, a lot of transformed material has been produced, could be of commercial interest and could therefore be unintentionally released on the market. The unintentional use of GM petunia in breeding programs has indeed recently been observed. This review lists scientific publications using GM ornamental plants and tries to identify whether these plants could be detected by molecular biology tools commonly used by control laboratories.

## Introduction

Ornamental plants are economically important for the horticultural industry^1^. They are sold all over the world, and used in gardening, landscaping, and floristry as cut flowers^2^. Genetic modification of ornamental plants has been used to introduce original traits of high commercial interest for the producers and/or consumers, such as improved floral anatomy and morphology, new floral color, induced early flowering, enhanced fragrance or longevity, stress tolerance or disease resistance^1–6^. At least 50 ornamental plants have been transformed, the main species including rose (*Rosa hybrida*), chrysanthemum (*Chrysanthemum morifolium*), petunia (*Petunia hybrida*), and carnation (*Dianthus caryophyllus*)^4^. However, very few genetically modified (GM) ornamental varieties have obtained the regulatory approval and have reached the market so far. Indeed, the authorization process required for such plants has a strong impact on the profitability of the development of such products. In the database of biotech/GM crops approval of the International Service for the Acquisition of Agri-biotech Applications (ISAAA), only three ornamental species are listed: petunia, rose, and carnation. Nineteen GM events have been registered in the ISAAA database for carnation, one GM event for petunia, and two GM events for rose. So far, only flower color-modified varieties of carnation and rose have been released on the market of some countries, depending on their regulation concerning production and/or commercialization of GMO^4,6^. In Europe, only two GM carnation varieties may currently be marketed as cut flowers. Genetic modification of ornamental plants has also commonly been used by research groups to study the genetics behind metabolic pathways, physiology, development, interactions with pathogens^7–11^. Numerous genetically transformed material of potential commercial interest have been produced. If by any mean, the traceability of such material was lost, it could be considered as non-GM and introduced in breeding programs.

In 2017, transgenic petunia plants were detected on the market in Europe and in the United States^6^. The detected GM events contained the A1 gene from *Zea mays* L. encoding the enzyme dihydroflavonol reductase, which was first introduced into a mutant petunia defective for this gene^12^. The first transformed plants had been obtained to study the biochemistry and genetics of the flavonoid metabolic pathway. These plants also had the particularity of producing flowers of an orange color not previously seen in the genus^12^. Further studies showed that differences in the methylation of the promoters occurring in the progeny of these plants conducted to the attenuation of the orange phenotype^13,14^. Such plants have never been through the authorization process required in the European Union and should not have been commercialized. Petunia plants containing the A1 gene have nevertheless been found on the market. Furthermore, the detected plants showed several phenotypes (unpublished data), suggesting that the transgenic material has been used in breeding programs for several years. In respect to the absence of approval for marketing or cultivation in Europe, numerous GM petunia plants had to be withdrawn from the European market in 2017.

The construct inserted in the genome of the detected GM petunia contained the A1 gene but also a 35S promoter (p35S) derived from the Cauliflower mosaic virus (CaMV). This promoter, with the nopaline synthase terminator from *Agrobacterium tumefaciens* (tNos), and to a lesser extent sequences such as the phosphinothricin acetyltransferase gene from *Streptomyces hygroscopicus* (bar) or the 5-enolpyruvylshikimate-3-phosphate synthase gene from *Agrobacterium tumefaciens* sp. strain CP4 (ctp2-cp4epsps), have been recurrently introduced in constructs used for genetic modification of plants. To test whether a sample may or not contain GM plant material, laboratories use these sequences as screening elements^15^. Diagnostic is mostly based on real-time PCR detection targeting the sequences of interest (p35s, tNos…)^16^.

The recent detection of transformed unauthorized petunia developed for research purpose in the 80’s demonstrates the risk that represents the potential non-intentional use of research plant material in breeding programs, especially when the transgene provides a trait of commercial interest. The aim of the present review was to assess the kind of GM ornamentals, which have been developed for research purpose and could therefore be found on the market if used involuntarily in breeding programs. Scientific publications related to research programs involving the transformation of ornamental species and for which the introduced trait could confer a commercial advantage compared with varieties on the market were listed. In addition, we investigated if such unauthorized material would be detectable by laboratories testing for the presence of GM plants.

## Literature studied

From previously published reviews and articles in database of peer-reviewed literature, 166 scientific publications related to genetic modifications of ornamental plants have been collected (Table 1). Among these publications, 29 ornamental plants were represented (Table 2); with chrysanthemum (26.7%), petunia (15.2%), orchidaceae (6.7%), rosa (6.7%), dianthus (5.5%), and torenia (5.5%) being the main ones (above 5%). Among the listed GM events, 88.5% were transformed via *Agrobacterium tumefaciens*, 9.1% via biolistic methods, and 3.0% via CRISPR/Cas9 (CRISPR: clustered regularly interspaced short palindromic repeats; Cas9: CRISPR-associated protein 9). Among these publications, 15 GM traits of potential commercial interest were identified relating to biotic/abiotic stress resistance and plant attributes (Table 1). Among these traits, the modification of the flower color was the most studied with 29.1% of the publications (Table 3). Other important GM traits were morphology (12.7%), longevity (12.1%), early flowering (8.5%), fungi and virus resistance (7.9%) (Table 3). Modification of ornamental plants with several traits was studied in 10.3% of the scientific publications.

**Table 1:**
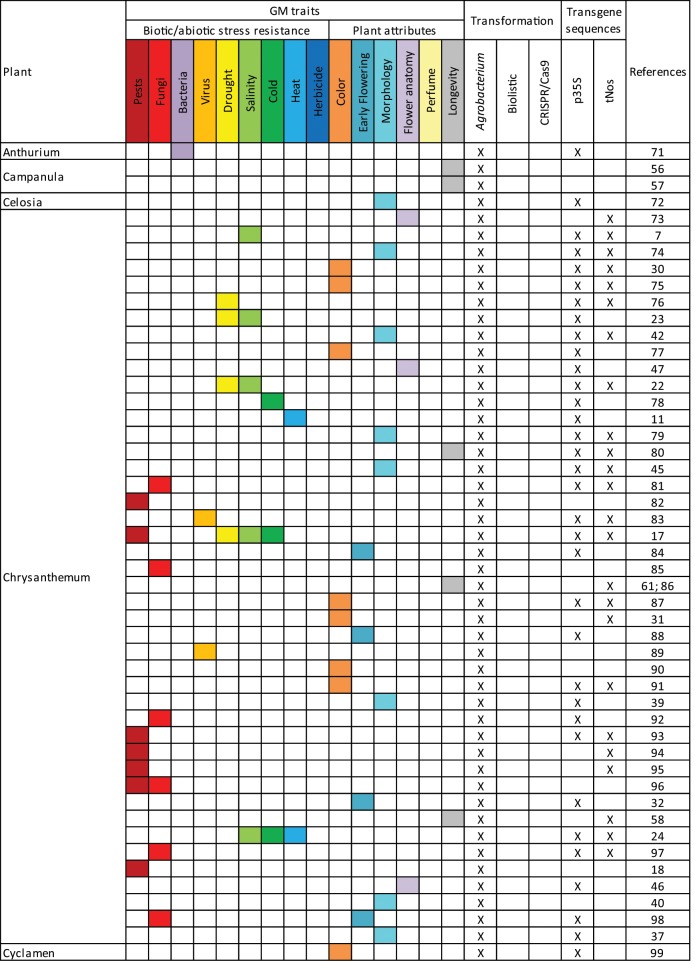

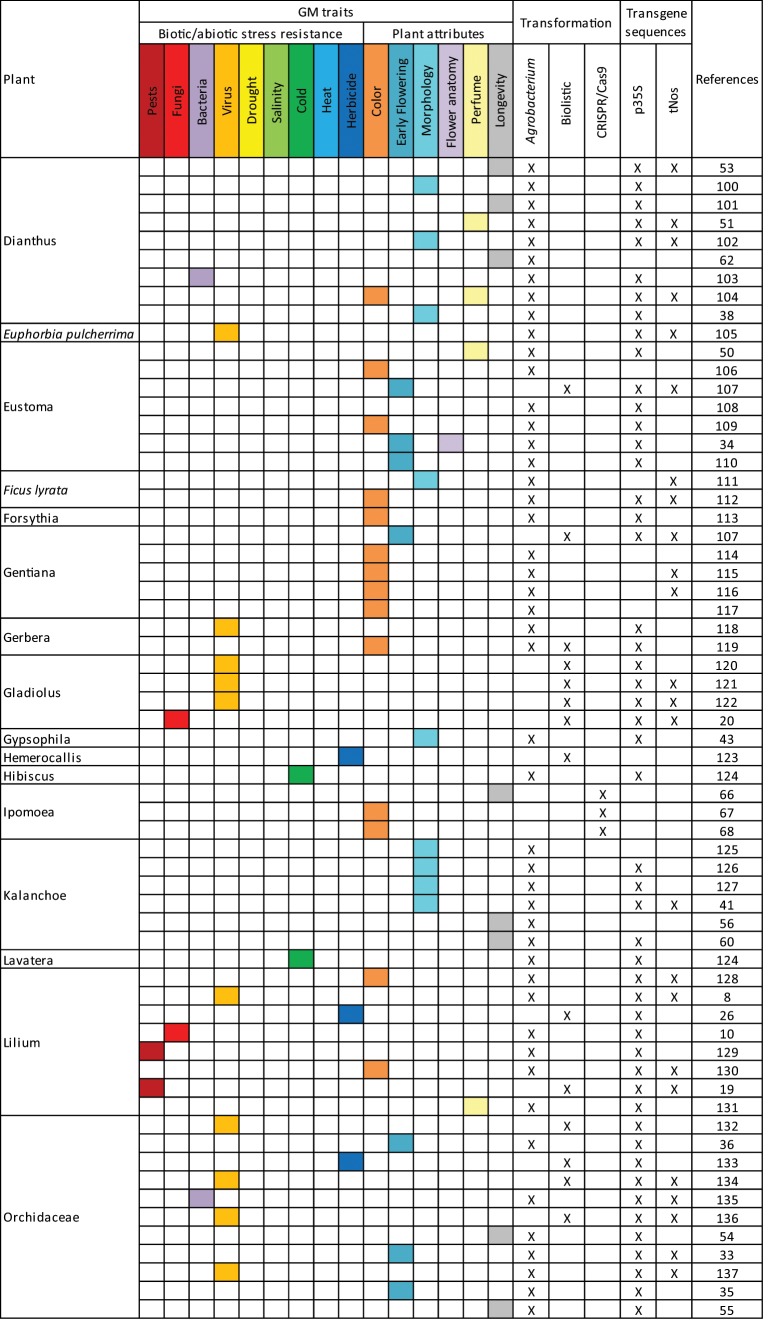

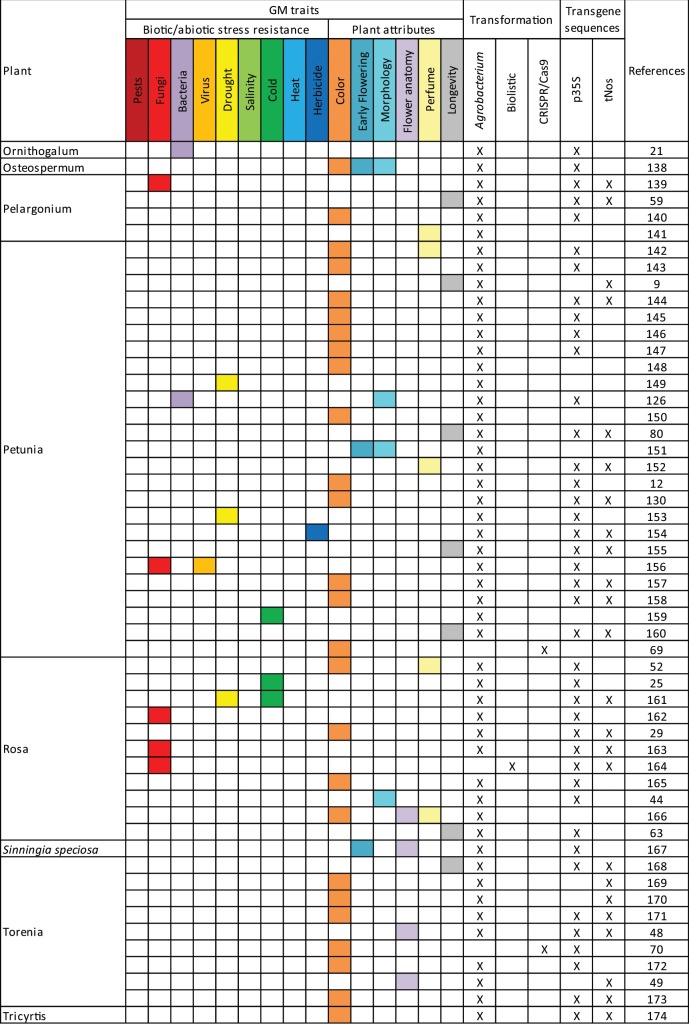
Overview of GM ornamental plants in scientific publications.

**Table 2:** Representation (%) of GM ornamental plants in scientific publications.

Plants	%
Anthurium	0.6
Campanula	1.2
Celosia	0.6
Chrysanthemum	26.7
Cyclamen	0.6
Dianthus	5.5
*Euphorbia pulcherrima*	0.6
Eustoma	4.2
*Ficus lyrata*	1.2
Forsythia	0.6
Gentiana	3.0
Gerbera	1.2
Gladiolus	2.4
Gypsophila	0.6
Hemerocallis	0.6
Hibiscus	0.6
Ipomoea	1.8
Kalanchoe	3.6
Lavatera	0.6
Lilium	4.8
Orchidaceae	6.7
Ornithogalum	0.6
Osteospermum	0.6
Pelargonium	2.4
Petunia	15.2
Rosa	6.7
*Sinningia speciosa*	0.6
Torenia	5.5
Tricyrtis	0.6

**Table 3:** Representation (%) of GM traits in scientific publications.

GM traits	%
Pest resistance	5.5
Fungi resistance	7.9
Bacteria resistance	3.0
Virus resistance	7.9
Drought resistance	4.2
Salinity resistance	3.0
Cold resistance	4.8
Heat	1.2
Herbicides	2.4
Color	29.1
Early flowering	8.5
Morphology	12.7
Flower anatomy	4.8
Perfume	5.5
Longevity	12.1
Several traits	10.3

## Overview of GM traits in ornamental plants

### Biotic stress resistance

Biotic stress occurs in plants due to damage done by plant pathogens or pests (bacteria, fungi, virus, insects, parasites, nematodes, aphids) during production, storage, distribution, and end-consumer use. Plant growth and yield are strongly influenced by biotic stress. Several ornamental species have been transformed to introduce or enhance resistance to pathogens or pests. Various genes coding for toxins, protease inhibitors, trypsin inhibitors, lectins, etc. have been employed to create pest resistance in plants^5^. As an example, increased resistance to aphids was observed in transgenic chrysanthemum overexpressing transcription factor CmWRKY48^17^ or the protease inhibitor Sea Anemone Equistatin (SAE)^18^. Expression of a cystatin transgene in *Lilium* *longiflorum* conferred resistance to the root lesion nematode *Pratylenchus penetrans*^19^. Resistance against bacterial and fungal diseases has been created by transferring various genes coding for chitinase, glucanase, osmotin, defensin, etc. into various ornamental plants^5^. Transgenic gladiolus expressing a synthetic antimicrobial peptide, D4E1, had an increased resistance to the soilborne fungus *Fusarium oxysporum* f. sp. *gladioli*^20^. The antimicrobial peptide tachyplesin I (tpnI) expression reduced bacterial proliferation, colonization, and disease symptoms (reduced by 95–100%) in transgenic ornithogalum plant tissues^21^. Virus resistance has been generally introduced by transferring coat protein gene of the virus in the plant. As an example, resistance to cucumber mosaic virus (CMV) was enhanced when a defective CMV replicase gene was transferred to the lilium^8^. Disease resistance in ornamental plants is greatly expected as disease symptoms on plants or cut flowers make the product unmarketable or unacceptable for export^4^.

### Abiotic stress resistance

Production of ornamental plants can be affected by abiotic stresses, such as drought, salinity, cold, heat, herbicides, etc. These stresses can affect plant growth and productivity as they generally damage the cellular machinery by inducing physiological, biochemical, and molecular changes in the plants^5^. Research on GM ornamentals has been conducted to improve abiotic stress resistance. Genes targeted for genetic transformation code for stress-protecting compounds have been classified into three categories: (i) genes coding the synthesis of various osmolytes such as mannitol, glycine betain, proline, and heat shock proteins, (ii) genes responsible for ion and water uptake and transport like aquaporins and ion transporter, etc., and (iii) genes regulating transcriptional controls and signal transduction mechanism, such as MAPK, DREBI, etc.^5^. As an example, overexpression of *AtDREB1A* gene increased drought and salt stress tolerance^22^, as does the overexpression of *ClCBF1*^23^ in transgenic chrysanthemum. The overexpression of the *Chrysanthemum crassum* plasma membrane Na + /H + antiporter gene *CcSOS1* improved the salinity tolerance of chrysanthemum plants^7^. A construct carrying both *CcSOS1* (from *Chrysanthemum crassum*) and *CdICE1* (from *Chrysanthemum dichrum*) was constitutively expressed in the chrysanthemum variety “Jinba”^24^. Improved sensitivity to low temperature, drought, and salinity was observed in transgenic plants as measured by visible damage and plant survival^24^. The *MtDREB1C* gene, isolated from *Medicago truncatula*, enhanced freezing tolerance in transgenic China rose without morphological or developmental abnormality^25^. Resistance to herbicide was obtained in transgenic *Lilium* *longiflorum* expressing the bialaphos-resistance gene PAT under the constitutive CaMV35S promoter^26^.

### Flower color modification

Flower color is one of the most important traits of ornamental plants influencing its commercial value. Flower color can also attract pollinators and protect floral organs^27^. Several ornamental plants have been engineered for flower color modifications by targeting flavonoids, anthocyanins, carotenoids, and betalains^28^. To create new flower color, biosynthetic pathways can be modified through the introduction of new genes, overexpression, or silencing of target genes^5^. The first ornamental plant genetically modified for flower color was an orange pelargonidin-producing petunia variety^12^. This was achieved by the expression of the A1 gene from *Zea mays* L. encoding the enzyme dihydroflavonol reductase in a petunia plant defective for this gene^12^. Other examples concerned roses, carnations, and chrysanthemum which lack violet/blue varieties, due to the absence of delphinidin-based anthocyanins^29^. This is attributed to their deficiency of flavonoid 3′,5′-hydroxylase (F3′5′H), a key enzyme in the synthesis of delphinidin^29^. Accumulation of delphinidin was achieved in roses or chrysanthemum by introducing the F3′5′H gene, turning the flower color purple or violet^29–31^. Blue chrysanthemum was produced by introducing the A3′5′GT gene encoding anthocyanin 3′,5′-O-glucosyltransferase, in addition to F3′5′H, into the host plant^31^.

### Early flowering

Flowering time is also an important trait of ornamental plants which can been engineered. Several reports have described successful gene introduction to produce flowers in comparatively short time, allowing the production of flowers at a lower cost^32^. MADS box genes constitute an example as they can control flowering time and floral organ development^3^. Overexpression of AP1-like genes, member of MADS box gene family, from Asteraceae induced early-flowering in transgenic chrysanthemum plants^32^. Transgenic Dendrobium orchids overexpressing DOAP1, an AP1 ortholog, displayed earlier flowering and earlier termination of inflorescence meristems into floral meristems than wild-type orchids^33^. Transgenic lisianthus plants, transformed with the MADS box gene OMADS1 from orchid flowered significantly earlier than non-transgenic plants^34^. Similarly, overexpression of MADS box genes, like DOSOC1 or OMADS1, promoted early flowering in transgenic orchids^35,36^.

### Morphology modification

Plant morphology can be engineered to provide economic advantages during commercial production of ornamental plants. Indeed, the manipulation of plant size and development can produce plants with uniform morphology, and reduce the use of chemical growth regulators thereby reducing production costs^37^. Genetic engineering of homeotic, Agrobacterium, phytochrome, and gibberellin genes has provided potential targets to modify and control plant growth and development^38^. A dwarf chrysanthemum was produced through heterologous expression of the mutant *Arabidopsis* gai (gibberellic acid insensitive) gene driven from its own promoter by decreasing gibberellin response^39^. Ectopic expression of a tobacco (*Nicotiana tabacum* L.) phytochrome B1 gene in chrysanthenum modified plant architecture; GM plants were shorter with larger branch angles than wild-type plants^37^. A miniature chrysanthemum was produced through RNAi expression vector targeting both DmCPD and DmGA20ox, which are related to brassinosteroids (BR) and gibberellins (GA) biosynthesis, respectively^40^. Production of compact plants of Kalanchoe was achieved by overexpression of homeotic genes KxhKN4 and KxhKN5^41^. The development of non-branching plants can reduce manual labor requirements during cultivation, decreasing the cost of flower production^42^. Transgenic gypsophila, carnation, or rosa exhibiting improved rooting were generated by expressing Agrobacterium rhizogenes ROL genes^38,43,44^. Enhancement of branching phenotype was observed in chrysanthemum by expression of the cytokinin biosynthesis gene ipt (isopentenyl transferase)^45^.

### Flower anatomy modification

Ornamental plants have been engineered to create new flower shapes that can increase their ornamental value. Flower formation involves the development of sepals, petals, stamens, and pistils, regulated by several genes involved in flower organ identity^34^. The overexpression of CmTCP20, a member of teosinte branched1/cycloidea/proliferating cell factors (TCPs) gene family, led to larger flower inflorescences and longer petals in chrysanthemum^46^. Overexpression of CmCYC2c in *Chrysanthemum lavandulifolium* led to significant increase in flower numbers and petal ligule length of ray florets^47^. Alteration of flower transition and formation was observed in transgenic *Eustoma grandiflorum* plants ectopically expressing the MADS box gene LMADS1-M from lily (*Lilium* *longiflorum*)^34^. Other studies have reported the modification of flower traits in transgenic torenia using chimeric repressors of Arabidopsis AGSRDX^48^ or TCP3^49^.

### Perfume modification

Floral scent volatiles are implicated in the evolutionary success of many plants as they attract pollinators and seed dispersers. Most fragrance compounds belong to three major groups: terpenoid, phenylpropanoid/benzenoid, and aromatic amino acid^3^. Most modern cut-flowers such as roses and carnations lack fragrance, probably because of the selection for other traits by breeders. Genetic engineering of ornamental plants could allow to transfer fragrance from one species to another, to induce new fragrance or enhance poor fragrance^4^. Fragrance in petals of transgenic lisianthus (*Eustoma grandiflorum*) was induced by overexpressing the *Clarkia breweri* BEAT gene (benzyl alcohol acetyl transferase) and fedding the transformed plants with alcoholic substrates^50^. Carnation plants were transformed with the linalool synthase (lis) gene from *Clarkia breweri* leading to the production of linalool and its derivatives in the transgenic plants^51^. In another study, an increase in the production of volatile phenylpropanoid/benzenoid compounds was observed in transgenic petunia flowers transformed with the Production of Anthocyanin Pigment1 (Pap1) Myb transcription factor from *Arabidopsis thaliana*^43^. Similarly, Pap1-transgenic rosa flowers showed increased levels of volatile phenylpropanoid and terpenoid compounds when compared with control flowers^52^.

### Longevity

Yellowing of leaves in ornamental plants, associated with senescence, have a negative attribute decreasing plant attractiveness, quality, and vase life^3^. In cut flowers, long vase life is a critical trait as they must survive several weeks before reaching the market^4^. Ethylene is a plant hormone, which plays a major role in the senescence process of plants^53^. Resistance to ethylene or inhibition of ethylene biosynthesis genes can increase shelf life^3^. Several ornamental plants have been transformed using genes encoding key enzymes of the ethylene pathway in order to inhibit senescence^53^. Expression of a mutated ethylene receptor gene etr1-1 from *A. thaliana* reduced ethylene sensibility in several ornamental plants, including orchid species^54,55^, *Campanula carpatica*^56,57^, chrysanthemum^58^, carnation^53^, *Pelargonium zonale*^59^, and *Kalanchoe blossfeldiana*^56,60^. Similarly, expression of a mutated ethylene receptor gene (mDG-ERS1s) reduced ethylene sensitivity in modified chrysanthemum^61^. Transgenic carnation plants containing an antisense ACC oxidase gene exhibited low ethylene production and delayed petal senescence^62^. An alternative approach to delay senescence in plants is to promote an increase in cytokinin levels^59^. Overproduction of cytokinins in petunia flowers transformed with PSAG12-IPT, enzyme which catalyzes the first step of the cytokinins biosynthesis pathway, delayed flower senescence, and decreased sensitivity to ethylene^9^. Delayed leaf senescence and enhanced resistance to exogenous ethylene were observed in miniature rose transformed with the same gene^63^.

### Detection of GM ornamentals

When available, information on the transgene has been taken into account to provide an indication of the detectability of the events using common molecular biology tools (Table 1). Among the constructs, at least 83.0% present one of the screening sequences p35S or tNos commonly targeted in GMO detection strategies and 35.8% present both. If tested, these plants would therefore be identified as genetically modified.

## Conclusion

The aim of this review was to list scientific publications related to GM ornamental plants developed by research groups and potentially presenting a commercial interest and to investigate if such unauthorized material would be detectable by laboratories testing for the presence of GM plants if unintentionally released on the marketplace. The goal was not to be exhaustive but to be as representative as possible of the constructs developed for research purpose. With 166 publications considered, it provides an overview of the constructs which could unintentionally reach the market without preliminary approval. The first conclusion of this review concerned the number of transformed ornamental species and the number of publications found per species. If many species have been transformed, two species of strong interest, chrysanthemum, and petunia, represent >40% of the collected data. With no consideration of any health, or environmental risk, of trade or commercial importance, these two species represent higher risks of unintentional use simply by the number of constructs available. The second outcome of the review is the ability of laboratories to detect at least 83.0% of the identified constructs.

This review also outlined the diversity of GM traits of potential commercial interest for the producers and/or consumers studied. Color modification dominates the GM ornamental research, however, since the first transgenic color-modified carnation variety released ~20 years ago, very few GM ornamental varieties have been field tested and have obtained the regulatory approval. Indeed, with the exception of color-modified varieties of carnation and rose, no transgenic varieties of flowers have reached the market so far. The cost and time required to obtain the regulatory approval are the major factors limiting the commercialization of such plants and the profitability of such developments.

Whole-genome sequencing of ornamental plants and the development of new molecular markers allow the identification of new genes of interest and related pathways^3^. Considering the ease of use of the new genome editing tools^64^ and especially of the CRISPR/Cas9 system^65^, more and more transformed material will be available. To our knowledge, there are five scientific publications related to successful genome editing of ornamental plants with trait of commercial interest, including ipomoea^66–68^, petunia^69^, and torenia^70^. CRISPR/Cas9-mediated mutagenesis of the EPHEMERAL1 locus delayed petal senescence in transformed ipomoea^66^. Flower color changes was induced using CRISPR/Cas9 technology^67,68,70^. As an example, ipomoea with pale-yellow petals was produced for the first time by successful knockout of InCCD4, involved in carotenoid degradation, using CRISP/Cas9 system^68^. Recently, the Court of Justice of the European Union (CJEU judgment of 25 July 2018, C-528/16) clarified that organisms obtained by new breeding techniques (NBT) are GMOs and fall within the scope of the EU GMO legislation. Meanwhile, several countries exporting crops to the EU (e.g., USA and Argentina) decided to not regulate such organisms as GMOs. Soon, it is probable that numerous varieties obtained by NBT will be available on the market outside of the EU, without any notification procedures in most countries. Accidental imports might occur between countries having different regulatory practices and policies regarding NBTs and environmental risk contamination should be considered.

Furthermore, groups will probably reduce the use of constructs including easily detectable promoters and terminators. The detectability of the produced transformed events will probably decrease in the near future; however, these considerations are not specific to ornamental plants.
